# Rethinking Hospital Sustainability: Circular Economy and Health Literacy: Comment on "A Review of the Applicability of Current Green Practices in Healthcare Facilities"

**DOI:** 10.34172/ijhpm.9297

**Published:** 2025-10-11

**Authors:** Patrizio Zanobini, Chiara Lorini, Guglielmo Bonaccorsi

**Affiliations:** Department of Health Sciences, University of Florence, Florence, Italy.

**Keywords:** Circular Economy, Health Literacy, Sustainability, Hospital

## Abstract

Climate change poses critical dangers to both environmental and human health, highlighting the need for sustainable healthcare strategies. In their recent paper, Soares et al explore how circular economy principles can be implemented in hospital settings in order to reduce environmental impact. This commentary expands on their work by highlighting a complementary but underexplored dimension: organizational health literacy (OHL). OHL emphasizes patient education and engagement, recognizing that informed and empowered patients are key to more efficient healthcare use. By enhancing patients’ understanding of their health conditions and care options, OHL helps prevent unnecessary procedures, tests, and hospital visits, thereby indirectly reducing resource consumption and supporting sustainable practices such as minimizing waste, conserving energy, and promoting telemedicine. In this way, OHL directly supports the operationalization of circular economy interventions described by Soares et al, ensuring their effectiveness and long-term sustainability. Merging OHL with circular economy practices represents a holistic pathway towards sustainable, equitable, and resilient healthcare delivery.

 The effects of climate change are far-reaching and complex, having profound consequences on the environment and human health. Increased temperatures, severe weather conditions, and ecosystem loss endanger biodiversity and ecological integrity, ultimately posing serious risks to human well-being. This interconnection is the core of the One Health model, an internationally recognized framework that highlights the interconnected relationships between human, animal, and environmental health.^1^ One Health model demonstrates how environmental disruptions, link to climate change and other factors, can lead to increased susceptibility to zoonotic diseases, food shortages, and diminishing living standards. Therefore, according to the World Health Organization (WHO), climate change represents one of the greatest threats to global health in the 21st century, reinforcing the urgent need for solutions that take into account human health together with environmental protection and animal well-being.^2^

 Driven by the ageing population, lifestyle related diseases, and advancements in medical technology and therapy, the healthcare sector has become a significant contributor of climate change. Globally, it is estimated that healthcare systems contribute approximately 4.4% to 5.2% to total greenhouse gas emissions, making them the fifth largest source of environmental pollution.^3^ As such, according to the paper of Soares et al^4^ to honour its commitment to first “do no harm,” the health sector is responsible for measuring and mitigating the environmental impact associated with healthcare. The authors present a broader conceptualization of the circular economy concept incorporating the 3Rs (reduce, reuse, recycle) and discuss its application in hospitals. The review identifies some domains of hospital activity in which principles of circular economy can be implemented and offers targeted intervention to reduce environmental impact. These domains include hospital design, waste management, energy and water consumption, transportation, travel and telemedicine, aimed at minimizing the environmental impact of healthcare-related mobility, green teams (dedicated to planning, training, and monitoring sustainability initiatives), food optimization, sustainable procurement, and staff behavior and engagement. Waste management and energy use emerge as the most frequently investigated topics, reflecting their fundamental importance to both environmental and operational concerns in hospitals.

 The review by Soares et al appears to be comprehensive, but it concentrates primarily on strategies for minimizing the environmental impact of healthcare services provided, and misses another key factor through which hospitals can reduce the demand for services and resources: the patient. A significant portion of healthcare resources is wasted because of the high prevalence of limited health literacy, which is estimated to affect one-third to nearly half of Europeans, nearly one in two adults in the United States, and an average of 55% of the population in Southeast Asia.^5^ Defined as the personal knowledge and competencies that enable people to access, understand, appraise and use information and services to promote and maintain good health, health literacy helps individuals in understanding complex health information and using this information effectively, thereby facilitating decision-making, self-care, and participation in health-related issues.^6^

 On the contrary, people with low health literacy engage in poor lifestyles and behaviors, as well as difficulties in accessing healthcare services, the latter frequently leading to poor adherence to care, delayed diagnosis, lack of engagement in preventive care, suboptimal management of chronic conditions, and inappropriate use of emergency services.^7,8^ In addition, the literature indicates that demands and expectations of patients result in overuse of healthcare services in a way that estimates suggest that as much as 30% of healthcare expenditure in the United States arises from preventable care.^6^ For instance, patients might insist on numerous diagnostic procedures and interventions even when clinical guidelines recommend against their adoption, contributing to worse health outcomes and increased healthcare resource consumption.^9^ As such, low health literacy levels constitute a key risk factor for excessive consumption of medical supplies, pharmaceuticals, and energy.^7,9^

 Since the beginning of the 2000s, the idea of organizational health literacy (OHL), defined as “the way in which services, organisations and systems make health information and resources available and accessible to people according to health literacy strengths and limitations” has been developed based on complex frameworks that identify main constituents and domains. One of the first papers presenting a framework for improving health literacy in healthcare organizations was published by Brach et al and was based on 10 key attributes.^10^ These attributes emphasized leadership commitment, workforce training, patient-centered communication, and navigation support, among others. Basically, by adopting these attributes, health-literate organizations reduce the need for high levels of patient health literacy, which indirectly benefits the environment. Empirical evidence suggests that OHL can reduce hospitals’ environmental impact through four main pathways. The first involves avoiding unnecessary utilization of services by educating patients about appropriate care use, preventive measures, and self-management strategies. This education directly reduces demand for healthcare services, thereby decreasing associated waste generation. Studies show that patients with adequate health literacy have 2.3 times fewer emergency department visits and lower hospital readmission rates, leading to substantial waste reductions from avoided procedures, diagnostic tests, and pharmaceutical consumption.^7^ The second pathway consists of streamlining care processes through clear communication and patient engagement, thus minimizing redundant or low-value procedures that drive unnecessary material consumption.^11,12^ Studies have demonstrated that access to comprehensible and pertinent clinical information through shared decision-making aids could prevent up to 20% of elective procedures.^13^ The third focuses on enhancing medication adherence and chronic disease self-management, which reduces wasted pharmaceuticals and prevents avoidable hospital readmissions.^8,12^ Finally, health-literate organizations prepare patients for safe home-based care through clear communication, tailored education, and caregiver training, while also integrating telemedicine technologies to provide remote monitoring and support. These strategies ensure continuity and quality of care, reduce hospital visits and resource-intensive stays, and lower the carbon footprint associated with patient and staff transportation.^14^ Patel et al found that telemedicine consultations can save an estimated average of 20 to nearly 100 kg of CO_2_ emissions per visit, mainly due to avoided patient travel.^15^

 Therefore, the combination of OHL and circular economy principles within hospitals is a promising path toward sustainability. By optimizing internal processes and educating patients on the appropriate use of services, hospitals can reduce resource consumption without compromising the quality of care. Furthermore, this dual commitment to environmentally sustainable practices and health literacy represents the foundation of the environmental health literacy, a concept which encompasses also other key areas such as environmental health, safety culture and risk communication. Environmental health literacy promotes a deeper understanding of how health is affected by various environment determinants while also encouraging proactive behaviors such as the application of circular economy principles and the advocacy for policies aimed at mitigating climate change.^16^ This perspective brings us back to the contribution of Soares et al, who highlight the importance of raising awareness across other sectors, given the widespread impact of pollution and environmental issues on public health. Promoting environmental health literacy among patients in hospital settings could serve as a bridge between the healthcare system and the population, fostering greater public responsibility regarding circular economy principles and creating an alliance with healthcare institutions to reduce their environmental impact. However, we are still far from achieving this goal. In fact, awareness and understanding of both health literacy and circular economy principles are still lacking even among healthcare professionals. As highlighted by Soares et al, the literature on the environmental sustainability of the hospitals is extremely limited, and this can partially account for the fact that one of the main challenges reported is the limited awareness and understanding among staff of the environmental impacts of healthcare practice. Although their analysis does not directly address OHL, this observation is equally relevant: without adequate staff engagement, training and understanding, efforts to integrate sustainability principles into OHL strategies are likely to encounter similar challenges. Behavioral change among healthcare professionals remains a key prerequisite for the successful implementation of both environmental and organizational transformations.

 Finally, it is important to remember that environmental sustainability, while crucial, represents only one dimension of a broader framework. Social and economic sustainability are equally essential to achieving equitable and robust healthcare delivery. Ignoring these components can lead to superficial, greenwashing-oriented strategies that ultimately harm the systems they aim to improve. A truly sustainable healthcare system requires an integrated approach, one that fosters innovation, promotes equity, and builds resilience, thereby enabling better health outcomes and a more sustainable future for both healthcare systems and the communities they serve.

## Disclosure of artificial intelligence (AI) use

 Not applicable.

## Ethical issues

 Not applicable.

## Conflicts of interest

 Authors declare that they have no conflicts of interest.
